# Phage therapy as a revitalized weapon for treating clinical diseases

**DOI:** 10.20517/mrr.2025.31

**Published:** 2025-09-24

**Authors:** Yingjie Wang, Yamei Yu

**Affiliations:** Department of Biotherapy, Cancer Center and State Key Laboratory of Biotherapy, West China Hospital, Sichuan University, Chengdu 610041, Sichuan, China.

**Keywords:** Phage therapy, multidrug-resistant bacterial infections, tumor therapy, phage display technology, artificial intelligence

## Abstract

The rising prevalence of multidrug-resistant (MDR) bacterial infections, coupled with the diminishing efficacy of antibiotics, has reinvigorated interest in bacteriophage (phage) therapy as a promising alternative, leveraging its unique bactericidal mechanisms and precise targeting capabilities. Concurrently, phage display technology has advanced tumor diagnostics and targeted drug delivery through high-throughput peptide screening. This review systematically evaluates the mechanisms, strategies, and clinical progress of phage-based applications in anti-infective and oncological therapies. Clinical evidence highlights its efficacy against respiratory, oral, wound, bloodstream, and urinary tract infections, alongside solid tumors. However, challenges persist, including limited host range, bacterial resistance, immunogenicity, inefficient delivery systems, and regulatory uncertainties. Future efforts should prioritize AI-driven phage optimization, standardized pharmacokinetic assessment, and interdisciplinary collaboration to accelerate clinical translation. Despite current limitations, phage therapy represents a transformative and scalable approach for combating antimicrobial resistance and advancing precision oncology, positioning it as a pivotal tool in addressing global health crises.

## INTRODUCTION

**Figure 1:**
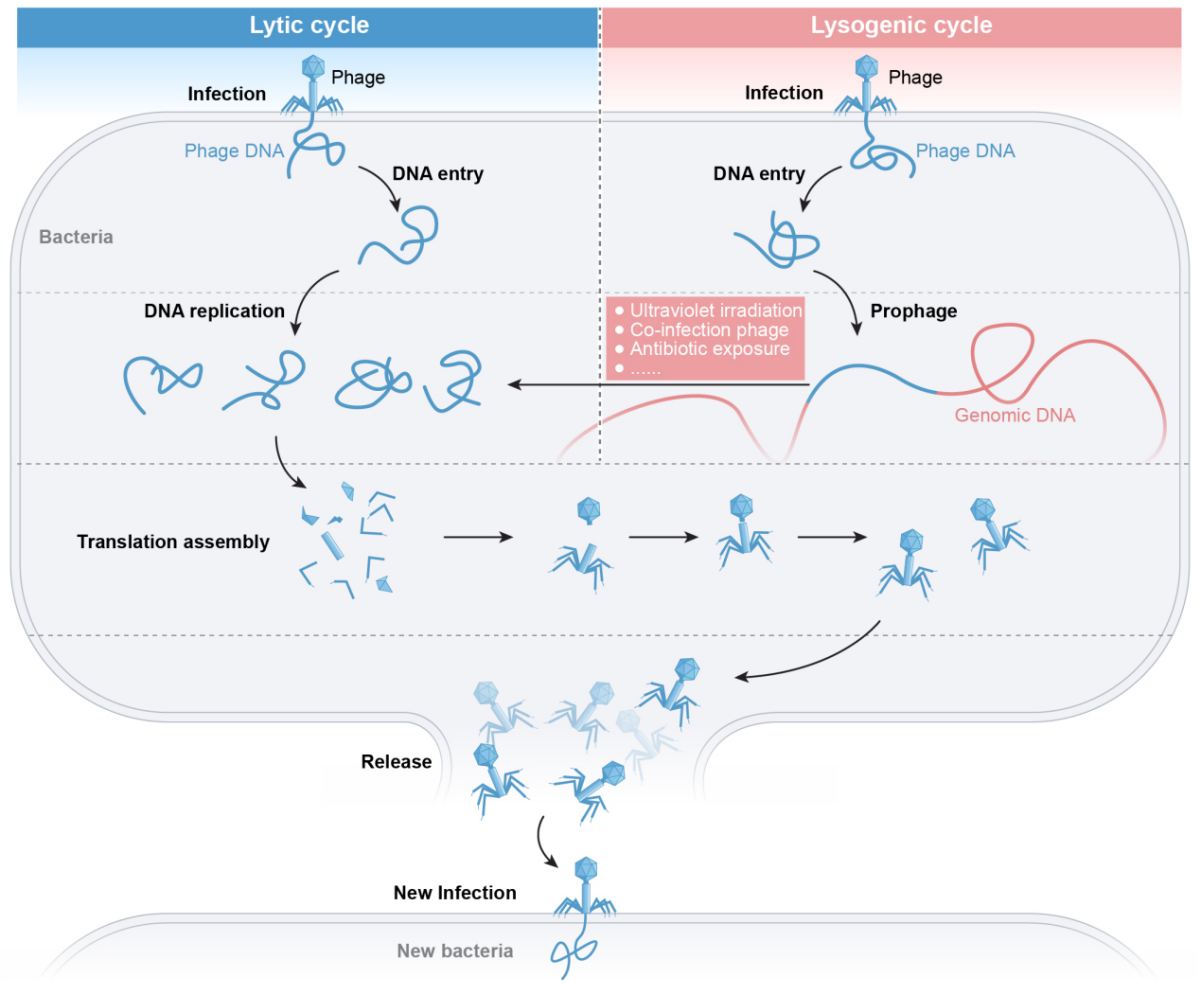
Stages of bacteriophage infection.

Phages are viruses that specifically infect and replicate within bacterial hosts, ultimately causing host cell lysis. First identified by Frederick Twort in 1915 and independently discovered by Félix D’Herelle in 1917^[1]^, phages typically range from 40-200 nm in size, with filamentous variants extending up to several micrometers. Their host range may be narrow or broad; for instance, phage JHP can infect multiple species [e.g., *Pseudomonas aeruginosa (P. aeruginosa)*, *Acinetobacter baumannii (A. baumannii)*, and *Escherichia coli (E. coli)*], whereas M13 demonstrates high specificity to *E. coli*^[2]^. Phage infection initiates upon binding to bacterial receptors located on cell walls, capsular polysaccharides, or flagella. The adsorption rate depends on host cell physiology, the phage’s mode of action, and local physicochemical variations in the surrounding medium^[3]^. Upon host interaction, phages typically follow one of two life cycles: the lytic cycle (virulent phages) or the lysogenic cycle (temperate phages). In the lytic cycle, virulent phages replicate inside the host, assemble progeny virions, and lyse the cell to release new particles. By contrast, temperate phages integrate their DNA into the host genome, where it replicates passively during host cell division^[4]^. Under certain environmental stressors, such as ultraviolet irradiation or antibiotic exposure, temperate phages may transition from lysogeny to the lytic cycle [Figure 1]^[4,5]^.

**Figure 2:**
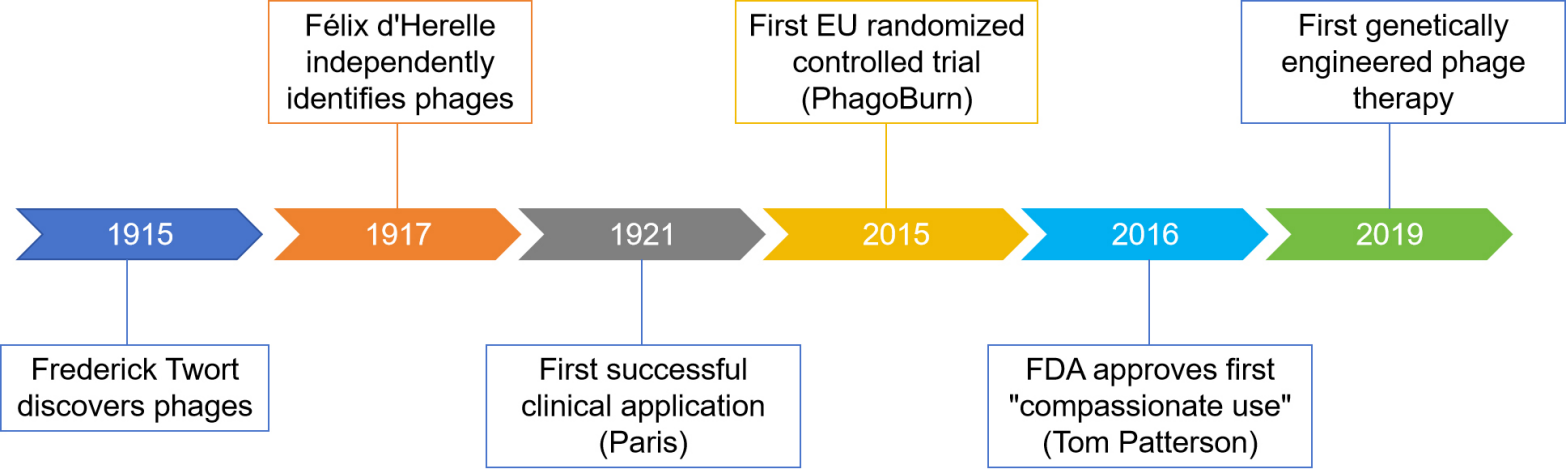
Key milestones in the development of phage therapy.

The therapeutic potential of phages was first demonstrated in 1921 with the successful treatment of dysentery in Paris^[6]^. However, interest in phage therapy declined during the mid-20th century with the advent of broad-spectrum antibiotics. In recent decades, the rapid rise of antibiotic-resistant infections, coupled with biotechnological advances that enable efficient identification and production of clinical-grade phages, has renewed interest in phage-based strategies as promising antimicrobial alternatives. Key milestones encompass the European Union’s first randomized controlled trial of phage therapy in 2015^[7]^, the first FDA-approved “compassionate use” phage treatment for Tom Patterson in 2016, and the first genetically engineered phage therapy developed via a U.S.-U.K. academic collaboration in 2019^[8]^ [Figure 2]. Between 2020 and 2024, 32 phage therapy clinical trials were registered worldwide on ClinicalTrials.gov. Beyond their role in combating bacterial infections, phages are increasingly recognized as modulators of bacteria-related pathologies, including gastrointestinal disorders, metabolic diseases, and, notably, tumor progression^[9]^.

## ALTERNATIVE TREATMENT FOR MDR BACTERIAL INFECTIONS

The escalating global burden of multidrug-resistant (MDR) bacterial infections has established them as the second leading cause of mortality worldwide, surpassed only by ischemic heart disease^[10]^. This crisis has catalyzed revived interest in phage therapy as a promising alternative, evidenced by a fivefold increase in PubMed-indexed clinical case reports over the past decade. Phage therapy offers distinct advantages as a precision antimicrobial, with reported efficacy rates of 50%-70%, an excellent safety profile, and no serious adverse events. Its key attributes include: strain-specific activity that preserves commensal microbiota; self-amplification at infection sites, which permits low-dose administration; and environmental sustainability as naturally occurring therapeutics^[11]^. Preclinical studies have demonstrated the efficacy of phages against major human pathogens, including *P. aeruginosa*, *E. coli*, *Klebsiella pneumoniae (K. pneumoniae)*, and *Achromobacter* species in both murine and clinical models^[12]^. Accordingly, phage therapy is now considered an effective weapon for eradicating MDR strains and combating refractory infections.

Phages mediate antimicrobial activity through three principal mechanisms^[13]^. First, lytic phages directly infect and lyse pathogenic bacteria via host-specific recognition and replicative cycles. Second, phage-mediated strategies can resensitize antibiotic-resistant bacteria to conventional antibiotics. For example, MDR efflux pumps, which confer resistance through active drug extrusion and reduced membrane permeability^[14]^, are evolutionarily co-opted by phages as entry receptors. This molecular exploitation enables phages to selectively target resistant populations, thereby enriching antibiotic-sensitive subpopulations and restoring therapeutic efficacy when combined with antibiotics. Liu *et al.* have also engineered phages to deliver antibiotic-sensitizing genetic elements into resistant hosts^[15]^. The enzymes expressed from these elements counteract resistance by degrading or sequestering antimicrobial compounds. Third, phages disrupt bacterial biofilms by penetrating extracellular matrices and binding selectively to bacterial receptors, thereby mitigating the ~ 1,000-fold increase in antimicrobial resistance associated with biofilms. Some phages also encode natural depolymerases that degrade bacterial surface polysaccharides, facilitating phage diffusion and subsequent bacterial lysis^[16]^.

**Figure 3:**
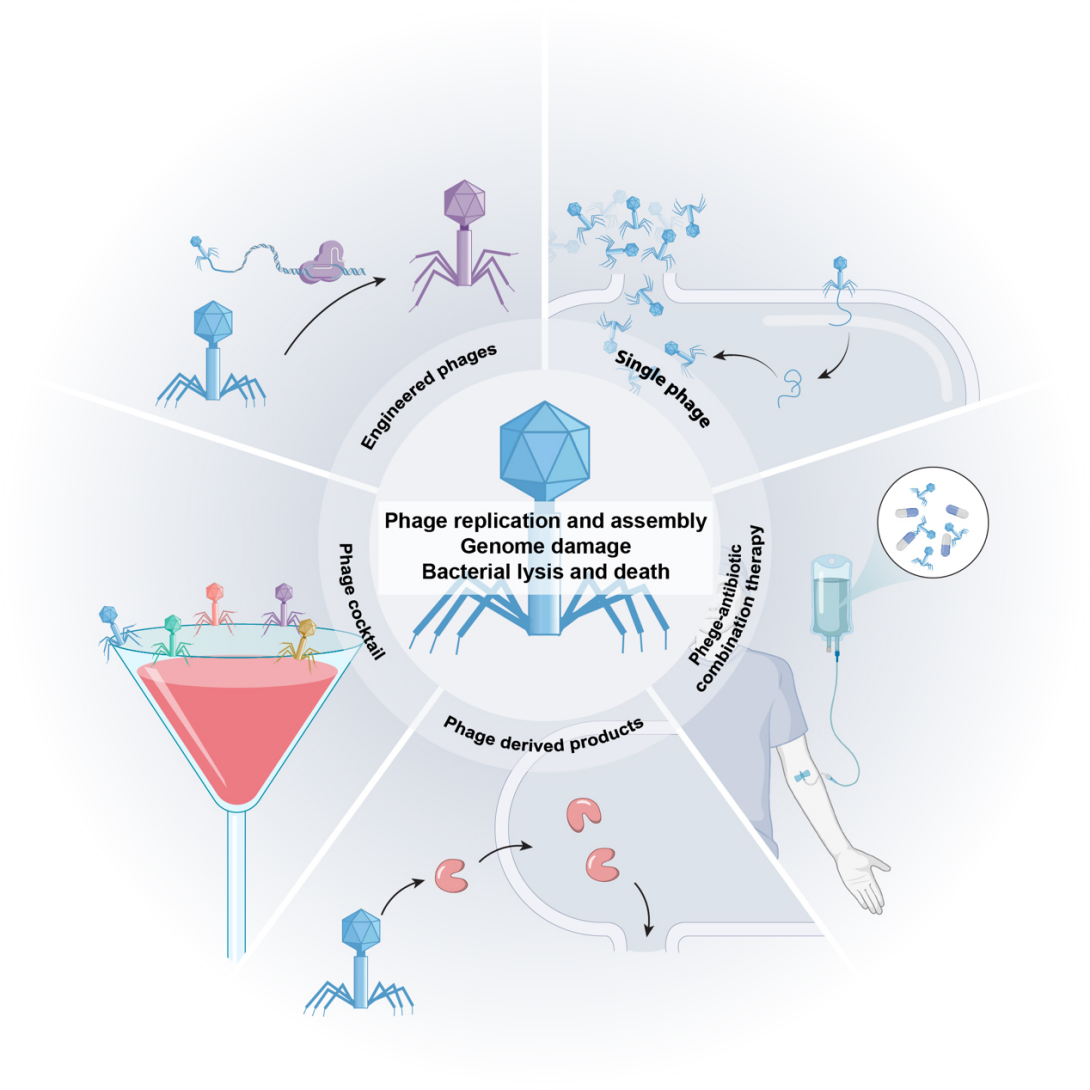
Therapeutic strategies of bacteriophages against bacterial infections.

Phage therapy can be tailored to infection characteristics using different treatment modalities [Figure 3]. For targeted infections involving well-defined pathogens (e.g., carbapenem-resistant *A. baumannii* [CRAB] pneumonia), monophage therapy enables precise eradication while conserving commensal microbiota^[17]^. However, monotherapy often rapidly selects for resistant variants, limiting its clinical utility. This constraint motivates rationally formulated phage cocktails that concurrently target diverse bacterial receptors or species, broadening therapeutic coverage while mitigating the likelihood of resistance^[18]^. Optimal phage cocktail formulation necessitates rigorous assessment of (i) host range breadth, (ii) lytic kinetics, (iii) formulation stability under physiological conditions, and (iv) clinical safety profiles, with particular emphasis on balancing maximal therapeutic efficacy against minimal off-target effects^[19]^.

Phage-antibiotic synergy (PAS) arises not only from phage-mediated restoration of antibiotic sensitivity, but also from enhanced phage replication in the presence of subinhibitory antibiotic concentrations^[20,21]^. For instance, a multicenter cohort study of 100 patients with diverse infections (pulmonary, integumentary, soft tissue, and osteoarticular) demonstrated 70% superior eradication rates with combination therapy compared to phage monotherapy^[22]^. However, these interactions exhibit significant complexity. Certain ribosome-targeting antibiotics can suppress phage replication by inhibiting virion assembly, thereby compromising therapeutic efficacy. Thus, the outcomes of phage-antibiotic combinations depend critically on treatment parameters - including dosage, frequency, timing, and administration sequence. Careful optimization of phage/antibiotic selection and mixing ratios is essential^[23]^, as these factors dictate antimicrobial synergy efficacy.

Beyond whole-phage therapy, bacteriophage-derived enzymes have emerged as promising alternatives with distinct clinical advantages, particularly endolysins and depolymerases. Unlike intact phages, these enzymes rarely induce bacterial resistance. Endolysins directly lyse bacterial cells by hydrolyzing peptidoglycan bonds in the cell wall, causing rapid osmotic rupture and death - a mechanism particularly effective against Gram-positive pathogens due to their exposed peptidoglycan layer. In contrast, depolymerases operate indirectly by enzymatically hydrolyzing surface polysaccharides, thereby exposing pathogens to host immunity rather than mediating direct bacteriolysis. Both endolysins and depolymerases demonstrate a narrow activity spectrum, enabling precise targeting of antibiotic-resistant pathogens while preserving commensal microbiota - a critical advantage over broad-spectrum antibiotics. Clinical studies indicate that endolysin-antibiotic combinations (e.g., semi-synthetic penicillin, vancomycin, or daptomycin) significantly reduce mortality in *Staphylococcus aureus (S. aureus)* bloodstream infections compared to antibiotic monotherapy^[24]^. Furthermore, engineered depolymerases could broaden strain coverage and delay resistance development when integrated into phage cocktails^[25]^. Importantly, although phage-derived enzymes follow conventional drug development pathways, advances in genomic sequencing and bioinformatics now allow for in silico enzyme design and recombinant production. This approach eliminates the need for phage cultivation, thereby accelerating clinical translation^[26]^.

Genetic engineering has significantly expanded the therapeutic potential of bacteriophages, enabling the development of customized phage libraries with enhanced capabilities. Targeted modifications include tail fiber mutations for host range expansion^[27]^, conversion of lysogenic phages into strictly lytic variants^[28]^, deletion of toxin genes for enhanced safety^[29]^, and incorporation of reporter genes for diagnostic applications^[30]^. These strategies allow researchers to engineer phages with precisely tailored properties. Engineered phages demonstrate broader activity spectra and enhanced stability against diverse bacterial strains, as evidenced by successful clinical applications in complex infections. A representative case involved a 15-year-old cystic fibrosis patient with extensively drug-resistant *Mycobacterium abscessus (M. abscessus)* infection. The patient achieved significant clinical improvement following treatment with a phage cocktail comprising both wild-type (Muddy) and engineered variants (ZoeJΔ45, BPsΔ33HTH-HRM10)^[8]^. While these advancements represent a major breakthrough in precision antimicrobial therapy, significant challenges remain. Genetic engineering may alter phage-host interactions, potentially diminishing infectivity or promoting resistance. Furthermore, unforeseen interactions with human cells or commensal microbiota may pose safety concerns. These risks necessitate rigorous mechanistic characterization and carefully controlled clinical evaluations as engineered phage therapies advance through structured translational pathways from laboratory development to phased clinical implementation.

Over the past two decades, phage therapy has evolved from an experimental concept into a clinical reality, as evidenced by a substantial increase in published case reports and controlled trials. Current clinical applications primarily target MDR infections across multiple systems, including respiratory, oral, wound, bloodstream, and urinary tract infections caused by pathogens such as *A. baumannii*, *P. aeruginosa*, *M. abscessus*, *K. pneumoniae*, *Enterococcus faecalis (E. faecalis) *, and *S. aureus*. The field has also established standardized protocols for personalized phage therapy, beginning with comprehensive isolation and phenotypic characterization of clinical strains, followed by rigorous phage screening through dual-layer plaque assays and whole-genome sequencing (WGS). Promising candidates undergo preclinical evaluation in animal models to assess safety and efficacy before advancing to clinical trials^[31]^. Recent case reports detailing phage therapy against MDR infections are summarized in Table 1.

**Table 1:** Current clinical cases of therapeutic phage applications

**Type**	**Causative pathogen**	**Phage**	**Outcome**	**Ref.**
Cystic fibrosis (CF)	*M. abscessus*	BPsΔ33HTH_HRM10 D29_HRM^GD40^	The positive rate of drug-resistant bacterial cultures decreased from 77.9% to 41.1% (*P* = 0.006), and the patient successfully underwent lung transplantation	[32]
	*A. xylosoxidans*	JWDelta, JWT 2-1	Although the pathogenic bacteria persisted in bronchoalveolar lavage cultures, patients showed progressive respiratory improvement	[33]
Chronic obstructive pulmonary disease (COPD)	*A. baumannii*	Ab_SZ3	Clinical improvement was evident by day 7, with negative bronchoalveolar lavage cultures and noticeable enhancement in lung function	[17]
Orthopedic surgeries	*K. pneumoniae*	KP1, KP2	The patient experienced pain relief, improved joint function, and no recurrence of infection during a 14-month follow-up	[34]
	*K. pneumoniae*	Kp_GWPB3, Kp_GWPA139	Pulmonary symptoms were alleviated, pleural effusion decreased, and fracture fixation was successful	[35]
IIIB open femoral fracture (IIIB-OFF)	*P. aeruginosa*	BFC 1.10	Complete infection resolution within nine months, enabling successful hip replacement	[36]
Burn wound (BW)	*A. baumannii*	PΦ-Bw-Ab	Demonstrated significant susceptibility to bacteriophages, indicating its potential as a targeted therapeutic agent	[37]
	*P. aeruginosa*	PP1450, PP1777, PP1792, PP1797	The patient’s critical condition improved, with enhanced quality of life	[38]
MDR bacteremia	*P. faecium*	Φ9184, ΦHi3	Phage therapy achieved significant clinical improvement and reduced intestinal colonization burden, though the patient ultimately died of pneumonia 7.5 months after phage treatment cessation	[39]
Urinary tract infections (UTI)	*E. coli*	EcAP29Φ234, EcAP29Φ237	Complete pathogen eradication was achieved three weeks after treatment, with urine cultures remaining negative four years later	[40]
	*K. pneumoniae*	a three-phage cocktail	Four weeks of intravenous phage therapy alone achieved complete resolution without recurrence	[41]

MDR: Multidrug-resistant.

### Phages in the treatment of respiratory diseases

Phage therapy has exhibited substantial clinical promise in addressing recalcitrant respiratory infections in cystic fibrosis (CF) patients, exemplified by a landmark case reported by Nick *et al*.^[32]^. This study detailed the treatment of a 26-year-old male CF patient (genotype H199Y/2184insA) with a persistent *M. abscessus* infection. Despite receiving intensive antibiotic regimens (≥ 4 concurrent drugs), the patient experienced repeated hospitalizations between 2016 and 2021. Laboratory analysis demonstrated susceptibility of *M. abscessus* isolated from sputum to two engineered phages (BPsΔ33HTH_HRM10 and D29_HRM^GD40^), prompting initiation of twice-daily intravenous therapy in March 2022 (1 × 10^9^ PFU and 1 × 10^8^ PFU, respectively). Treatment produced marked clinical efficacy with excellent tolerability and no adverse events. After initiation, microbiological outcomes demonstrated a statistically significant reduction in culture positivity from 77.9% to 41.1% (*P* = 0.006), declining further to 10% (*P* < 0.0001) over the subsequent 280-day period. This successful microbial control enabled the patient to undergo lung transplantation, resulting in substantial quality-of-life improvement. Phage therapy has also proven successful in managing persistent *M. abscessus* infections in chronic obstructive pulmonary disease (COPD). A notable case involved an 88-year-old COPD patient who developed respiratory failure secondary to carbapenem-resistant *Acinetobacter baumannii* (CRAB) infection. Phage therapy was administered via a vibrating mesh nebulizer every 12 h for 16 days, with progressive dose escalation. Concurrent antibiotics were maintained during the first 10 days. By day 7, clinical improvement was observed, as evidenced by sterile bronchoalveolar lavage (BAL) cultures and a significant enhancement in lung function^[17]^.

*M. abscessus* persists within host cells and forms biofilms^[42]^. In nutrient-deprived microenvironments of damaged lungs, these mycobacteria can enter a non-replicating “persister” state, enabling extended survival under hypoxic conditions while evading antibiotic activity^[43]^. The engineered phage BPsΔ33HTH_HRM10, originally temperate, was converted into an obligately lytic variant through partial deletion of its repressor genes, ensuring direct bacterial lysis upon infection. Meanwhile, host-range modification of D29_HRM^GD40^ enhanced its activity against clinical *M. abscessus* isolates. Phage-encoded lysozymes degrade *M. abscessus* biofilms, enabling direct access to bacteria for lysis. However, during prolonged antibiotic-phage therapy, neutralizing antibody production may compromise efficacy by inactivating phage particles. *In vitro* experiments demonstrated that although the activity of BPsΔ33HTH_HRM10 was significantly reduced by neutralization by day 149, D29_HRM^GD40^ retained sustained activity. Thus, using a dual-phage combination may mitigate the risk of resistance.

Phage therapy for *K. pneumoniae* infections has also achieved significant advances since its first documented application in 1991^[44]^. Between 2019 and 2023, twelve clinical trials evaluated phage therapy against MDR *K. pneumoniae* pneumonia, demonstrating clinical efficacy. In a representative case, Li *et al.*^[35]^ described a 54-year-old polytrauma patient with MDR *K. pneumoniae* pneumonia, concomitant brain and pulmonary contusions, and multiple fractures. Two courses of nebulized phage therapy (Kp_GWPB35 and Kp_GWPA139) yielded marked clinical improvement: resolution of pulmonary symptoms and pleural effusion facilitated definitive fracture fixation. While phage resistance emerged in some cases, it was often accompanied by reduced bacterial virulence. This suggests that even when complete bacterial clearance is not achieved, phage therapy may convert infections into more manageable forms.

Another exemplary case involved a 70-year-old male with recurrent *K. pneumoniae* infection following multiple orthopedic surgeries (reverse shoulder arthroplasty and pelvic fracture repair). Chronic pulmonary abscess infections represent refractory lesions with challenging clinical management, with no standardized therapeutic approach available. Because the bacteria persist in a phenotypic state, deep infections are particularly resistant to eradication by conventional antibiotics^[45]^. In the present case, the patient received sequential treatment with phages KP1 and KP2 in combination with ertapenem. This strategy was based on the understanding that monotherapy with a single phage may rapidly induce resistance, whereas combinations of phages can lead to competition and mutual inhibition^[46]^. Ultimately, the patient experienced pain alleviation and improved joint function, with no recurrence of infection during a 14-month follow-up. In addition, the regimen employed Hickman catheters for repeated administration, enabling direct delivery of high drug doses to the infected prosthetic site and thereby enhancing the overall therapeutic efficacy^[34]^.

Phage therapy has demonstrated clinical safety and therapeutic promise for controlling MDR bacterial colonization in both lung transplant candidates and recipients. Its peri-transplant application may expand eligibility for patients currently excluded at many centers, thereby reducing waitlist mortality and post-transplant infection risk^[47]^. Clinical evidence includes a 12-year-old cystic fibrosis patient who underwent two courses of phage therapy for extensively drug-resistant Achromobacter xylosoxidans infection. Although BAL cultures initially remained positive, the patient exhibited progressive respiratory improvement culminating in complete microbial eradication, with sustained clearance for over two years after discontinuation of phage and imipenem therapy^[33]^. In another report, Aslam *et al.* evaluated three transplant recipients with MDR infections, yielding variable outcomes^[48]^: two patients with airway complications and refractory *P. aeruginosa* pneumonia achieved clinical improvement and ventilator weaning, while a third patient with recurrent *Burkholderia dolosa (B. dolosa)* infection exhibited temporary radiographic and clinical improvement before relapsing and ultimately succumbing to disease recurrence. Notably, all cases maintained an exemplary safety profile without phage-related adverse events.

The development of phage-based therapeutics for respiratory diseases is advancing rapidly, with multiple candidates in clinical development. AP-PA02 (Armata Pharmaceuticals), a defined phage cocktail targeting *P. aeruginosa*, demonstrated favorable safety and tolerability in a phase 1b/2a trial involving patients with CF or non-CF bronchiectasis. Following the 2020 FDA approval of its investigational new drug application, the trial demonstrated sustained bacterial load reduction along with favorable pharmacokinetics, indicating effective lung targeting and minimal systemic exposure. These results prompted the initiation of a phase 2b trial (NCT05616221). In parallel, the Portuguese biopharmaceutical company TechnoPhage is developing TP-122A, an inhaled three-phage formulation targeting *P. aeruginosa* for ventilator-associated pneumonia. Currently under evaluation in a phase 1/2a trial (*n* = 15 adults), this nebulized formulation is designed to achieve efficient pulmonary delivery while evaluating safety and tolerability (NCT06370598).

### Phages in the treatment of refractory wound infections

Phage therapy represents a promising therapeutic strategy for refractory wound infections, including traumatic, surgical, and burn-related wounds. Traumatic wounds present substantial clinical challenges due to their heterogeneous wound dimensions, high risk of contamination, and frequent polymicrobial infections involving *E. coli*, *K. pneumoniae*, *A. baumannii*, *P. aeruginosa,* and *S. aureus*^[49-51]^. Bhartiya *et al.* conducted a prospective comparative trial evaluating bacteriophage therapy (BPT) versus conventional antibiotic therapy (CAT) in 54 patients with extensive traumatic wounds (> 36 cm^2^)^[52]^. The BPT group showed significantly improved outcomes: Higher sterile healing rates (100% *vs.* 20%; *P* < 0.001) at 14 days; Reduced mean healing time (28 days shorter than CAT); Shorter hospitalization (median 20 *vs.* 40 days; *P* = 0.002); and 67% lower treatment costs^[52]^.

A representative case demonstrating the potential of phage therapy involved a patient with type IIIB-OFF complicated by MDR *P. aeruginosa* osteomyelitis with fistula formation. *P. aeruginosa* infections are difficult to treat and have high recurrence rates^[53,54]^. Initial empirical antibiotic therapy failed to eradicate the infection and induced kidney injury. Further investigation revealed that the patient’s MDR *P. aeruginosa* strain had strong biofilm-forming capacity, rendering conventional antibiotics ineffective. Phages, with their ability to self-replicate and produce polysaccharide depolymerase, are uniquely suited to combat biofilm-associated *P. aeruginosa* infections^[55]^. As the isolate remained sensitive to ceftazidime-avibactam, the treatment strategy combined local administration of a *P. aeruginosa*-specific phage cocktail (BFC 1.10) with intravenous ceftazidime-avibactam and surgical debridement. Nine months later, the infection was eliminated, and the artificial hip joint was successfully implanted. Notably, the combined treatment of local phages and intravenous antibiotics promoted the eradication of infection in the proximal femur and pelvis, which was crucial for pseudoweight-bearing reconstruction^[36]^.

Surgical site infections (SSIs), defined as infections occurring within 30-90 days post-procedure at incision sites, represent some of the most common healthcare-associated infections^[56]^. These are typically caused by endogenous pathogens, with methicillin-resistant *Staphylococcus aureus* (MRSA) being the predominant etiological agent in contemporary surgical settings^[57]^. SSI management is complicated by bacterial biofilm formation, particularly resilient phenotypes producing extracellular polymeric substances (EPS). Biofilm-associated infections present a therapeutic challenge where phage therapy shows promise through its biofilm-penetrating activity and EPS depolymerization. Nadareishvili *et al.* conducted a retrospective case series of four complex postoperative infections caused by MDR pathogens, including chronic osteomyelitis (*n* = 2), diabetic foot infection (*n* = 1), and skin graft infection (*n* = 1)^[58]^. All patients achieved successful outcomes, with phage preparations (administered in various combinations) effectively improving health status and accelerating wound healing.

Burn injuries constitute severe trauma, as the impaired integumentary barrier predisposes patients to bacterial colonization and progression to severe wound infections. This challenge is further exacerbated by the prevalence of MDR pathogens, frequently causing life-threatening systemic complications such as sepsis and systemic inflammatory response syndrome (SIRS)^[59]^. In a prospective cohort study of 50 burn wound infections, Torabi *et al.* identified extensively drug-resistant *Acinetobacter baumannii* (XDR-AB) as the predominant pathogen in burn units, which demonstrated notable susceptibility to bacteriophage PΦ-Bw-Ab, indicating its potential as a targeted therapeutic agent^[37]^. The therapeutic potential of phage therapy was further highlighted in a critical case involving a patient with 85% total body surface area (TBSA) burns complicated by MDR *P. aeruginosa* infections, presenting as recurrent ventilator-associated pneumonia, skin graft infections, and bacteremia. A personalized treatment combining nebulized and intravenous phage therapy with immunostimulation (interferon-γ) and imipenem-relebactam resulted in complete resolution of the critical condition and substantial quality-of-life improvement^[38]^.

### Phages in the treatment of bacteremia

Accumulating preclinical evidence indicates the therapeutic efficacy of phage therapy against MDR bacteremia. Murine models have demonstrated complete eradication of XDR-AB bacteremia following intravenous lytic phage administration^[60]^. Clinical evidence comprises a representative case of a 57-year-old female with recurrent *Enterococcus faecium (E. faecium)* bacteremia^[39]^. *E. faecium* is resistant to many first-line antibiotics and often causes persistent or recurrent infections. The patient eventually developed resistance to combined antibiotic treatment over seven years. *In vitro* time-kill assays demonstrated that phage-antibiotic combinations achieved superior bacterial eradication. Subsequently, the patient received combined treatment with two lytic phages (Φ9184 and ΦHi3), with doses escalating from 1 × 10^9^ PFU per dose three times daily to 2 × 10^9^ PFU per dose. Following the integration of phage therapy into her antimicrobial regimen, clinical symptoms continued to improve over several months, and the intestinal burden of *E. faecium* was significantly reduced. However, the patient died from pneumonia 7.5 months after discontinuation of phage treatment, potentially attributable to the emergence of anti-phage neutralizing antibodies. Overall, these findings not only underscore the potential value of phage therapy in treating MDR bacteremia but also reveal limitations in its application.

### Phages in the treatment of urinary tract infections

Phage therapy has demonstrated significant potential in managing recurrent urinary tract infections (UTIs), particularly in high-risk populations such as kidney transplant recipients (KTRs). These patients frequently develop UTIs associated with persistent colonization of intestinal and urinary microbiota, where CAT often fails to prevent recurrences^[61,62]^. A documented case involved a 17-year-old female KTR with recurrent urosepsis caused by ESBL (extended-spectrum beta-lactamase)-producing *E. coli*. Over four months, she required four hospital admissions due to repeated infection events. During treatment, she received successive courses of ertapenem and underwent fecal microbiota transplantation (FMT) to decolonize ESBL-producing *E. coli* in the intestine. Despite these interventions, urosepsis recurred repeatedly, and the source of the urinary ESBL-producing *E. coli* after antibiotic therapy remained undetermined. Ultimately, the patient was treated with intravenous injections of a combination of two unrelated phages (EcAP29Φ234 and EcAP29Φ237) alongside antibiotics. After three weeks, continuous microbial eradication was achieved, and sterile urine cultures were maintained throughout a 48-month follow-up. Notably, using a combination of phages rather than a single strain effectively countered bacterial resistance, which is particularly advantageous for immunocompromised patients^[40]^.

Single-phage therapy has also proven effective in certain patient cases. A KTR with bacteriuria caused by ESBL-producing *K. pneumoniae* achieved complete clinical resolution following a four-week intravenous phage monotherapy, with no recurrence observed during a 12-month follow-up^[41]^. These cases highlight phage therapy’s dual role as both an adjunct to antibiotics and a standalone treatment for complex UTIs. In parallel, pharmaceutical development of phage-based therapies for UTIs has advanced significantly. Locus Biosciences’ LBP-EC01 has emerged as a leading candidate. This CRISPR-enhanced phage cocktail, comprising six phages, specifically targets antibiotic-resistant *E. coli* strains. Early results from the ongoing Phase 2/3 ELIMITATE trial, initiated in July 2022, are promising^[63]^. Preliminary data show rapid bactericidal activity, with a significant reduction in urinary *E. coli* load within four hours of administration, followed by complete resolution of UTI symptoms by day 10 in all evaluated patients. These clinical developments position phage therapy as a transformative solution for UTIs, particularly those caused by MDR pathogens, where conventional options are limited.

### Phages for treating oral infections

Research has highlighted the potential of phage therapy in targeting two prevalent oral infections: periapical periodontitis and dental caries. In periapical periodontitis, studies have focused on combating antibiotic-resistant *E. faecalis* biofilms, which often reduce the effectiveness of root canal treatments. The enterococcus-specific phage HEf13 has shown strong biofilm-disrupting activity, both by preventing biofilm formation and by degrading established biofilms. Moreover, combining HEf13 with chlorhexidine or penicillin produced synergistic effects, significantly enhancing antibacterial activity compared with either agent alone^[64]^. For dental caries caused by *Streptococcus mutans (S. mutans)*, phage SMHBZ8 has demonstrated preventive efficacy *in vitro*, inhibiting carious lesion formation, and *in vivo*, suppressing caries progression in murine models, as confirmed by Micro-Computed Tomography (μCT) scanning^[65]^.

## ALTERNATIVE TREATMENT FOR TUMORS

Phage applications have now expanded beyond conventional antimicrobial functions, demonstrating therapeutic potential in oncology. This shift is supported by growing evidence linking specific bacterial populations to tumor progression^[66]^, along with historical observations of phage accumulation in neoplastic tissues and their capacity to inhibit tumor growth^[67]^. A seminal advancement was made by Zheng *et al.*, who developed an innovative phage-based precision strategy targeting *Fusobacterium nucleatum (Fn)* in colorectal cancer^[68]^. Their approach combined a carefully selected P1 phage with irinotecan-loaded glucan nanoparticles (IDNPs), generating a hybrid nanosystem that enhanced tumor-specific drug accumulation and significant *Fn* suppression while preserving commensal microbiota^[68]^. Beyond their antimicrobial activity, phages also exhibit notable immunomodulatory effects, as exemplified by the M13 phage, which activates the TLR pathway via its CpG-rich genome, thereby stimulating antigen-presenting cells and enhancing antitumor immune responses^[69]^.

**Figure 4:**
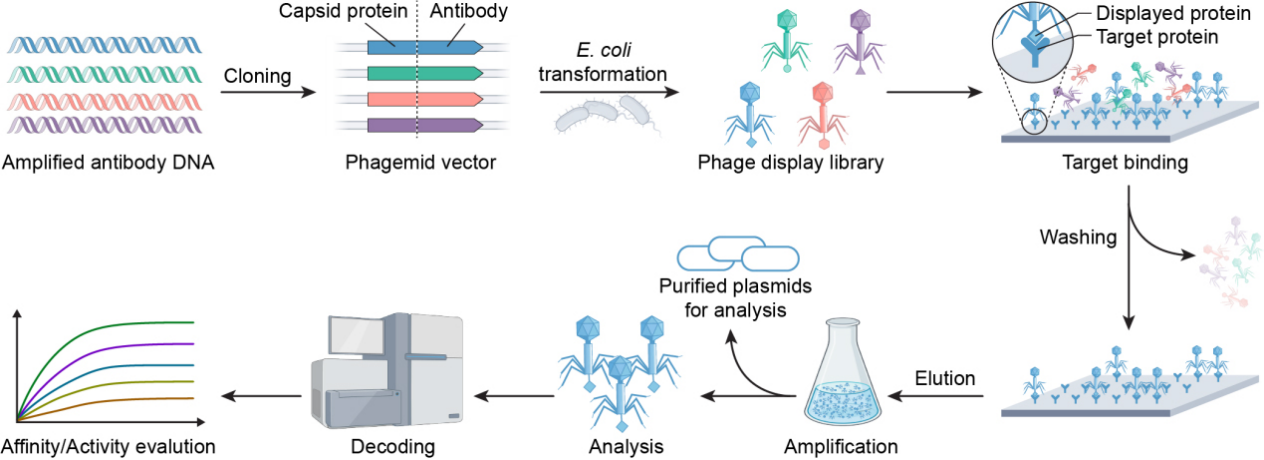
Schematic representation of phage display technology.

In parallel, phage display technology has contributed significantly to tumor treatment, bringing hope to patients. This technology, developed by Smith in 1985^[70]^, has revolutionized diverse fields, including peptide screening for cancer diagnosis^[71]^, drug development^[72]^, optimization of drug delivery systems^[73,74]^, material modification^[75]^, and studies of protein-ligand interactions or binding sites^[76]^. Its versatility arises from several key advantages: exceptional library diversity (typically 10^9^-10^11^ clones), cost-effectiveness relative to conventional methods, and compatibility with diverse targets ranging from proteins to inorganic materials. Phage display involves the insertion of exogenous DNA fragments into phage capsid protein genes, resulting in the surface display of functional polypeptides while maintaining viral infectivity. The standard screening process^[77]^, illustrated in Figure 4, includes: (1) construction of diverse peptide libraries; (2) incubation with target molecules; (3) stringent washing to remove non-specific binders; (4) elution of target-bound phages; and (5) amplification through 3-5 rounds of biopanning to enrich high-affinity clones. Selected phage clones are then identified by sequencing the peptide-encoding region^[78]^. The filamentous M13 phage remains the preferred vector due to its unique structural plasticity and high biological stability.

Despite its strengths, traditional phage display faces several inherent limitations: (1) selection bias favoring phages with optimal replication rather than optimal binding; (2) restricted library diversity due to bacterial transformation bottlenecks; and (3) time-consuming iterative screening. Cutting-edge innovations are addressing these challenges through three key strategies: (1) engineered libraries incorporating non-canonical amino acids to dramatically expand chemical diversity^[79-81]^; (2) NGS-powered deep sequencing for quantitative analysis of entire phage populations in a single experiment^[82]^; and (3) microfluidic high-throughput platforms that automate and miniaturize the screening workflow. These synergistic advances are collectively enhancing the precision and throughput of phage display technology, solidifying its position as a cornerstone of modern cancer drug discovery^[83,84]^.

### Phage display in diagnosis

Phage display technology enables the identification of highly specific peptide ligands, facilitating early cancer detection via femtomolar-level biomarker recognition^[78]^. Site-directed mutagenesis of M13 filamentous phage coat proteins (particularly the pIII and pVIII domains) produces robust antibody mimetics, enabling direct biomarker detection in complex biofluids (serum/plasma) while reducing false positives and low sensitivity inherent to conventional immunoassays^[85]^. Peptides offer notable advantages as targeting agents, including high binding affinity (typically in the low nanomolar range)^[86]^, efficient cellular internalization^[87]^, low immunogenicity^[88]^, and favorable pharmacokinetics - characterized by rapid clearance from non-target tissues and sustained retention in malignant lesions^[89]^.

Tumor-targeting peptides, including radiolabeled, bioluminescent, fluorescently labeled, and nanomaterial-based peptides, have emerged as powerful diagnostic tools for detecting malignancies, as detailed in Li’s review^[90]^. Phage display-derived peptides for *in vivo* molecular imaging primarily target tumor-overexpressed proteins or vascular-associated components, exemplified by the HER2-binding KCCYSL and the galectin-3-targeting peptide ANTPCGPYTHDCPVKP, both of which have been applied in imaging studies^[91,92]^. Galectin-3-targeting peptides, derived from a cysteine-restricted library developed by George Smith’s lab, exhibit remarkable biological activity by inhibiting approximately 50% of tissue factor-galectin-3 interactions. Beyond direct targeting, cell-penetrating peptides (CPPs) can deliver diagnostic radioisotopes, such as ^18^F-labeled RGD peptides, which selectively accumulate in integrin-overexpressing tumors via integrin-dependent endocytosis^[93,94]^. Phage display-derived 2rs15d nanoparticles also exhibit high specificity for recombinant HER2 protein and HER2-positive cells. This nanoplatform exhibits optimal pharmacokinetic properties for breast cancer imaging, with high tumor specificity and rapid blood clearance^[95]^.

### Phage display in drug development

Phage display technology has become a cornerstone of drug discovery, enabling the identification of numerous therapeutic peptides, cyclic peptides, and over 10 commercially available antibody drugs, with many others in development. Its key advantages include the ability to generate molecules with low off-target toxicity, efficient renal clearance, high target specificity, and enhanced cellular delivery^[96]^. This approach has produced several clinically approved therapeutic antibodies with demonstrated efficacy across diverse diseases.

In oncology, Programmed Death-Ligand 1 (PD-L1) inhibitors (atezolizumab^[97]^ and avelumab^[98]^) have shown clinical efficacy in cancer immunotherapy. Other notable examples include ramucirumab^[99,100]^ for advanced gastric cancer and necitumumab^[101,102]^ for squamous non-small-cell lung carcinoma. Phage display-derived drugs have also been successfully applied to autoimmune disorders, including belimumab^[103]^ for systemic lupus erythematosus and lupus nephritis, and ixekizumab^[104,105]^ and guselkumab^[106]^ for psoriasis. In hematology, therapeutic antibodies include Moxetumomab^[107,108]^ for hairy cell leukemia, caplacizumab^[109,110]^ for thrombotic thrombocytopenic purpura, and emapalumab^[111,112]^ for hemophagocytic lymphohistiocytosis. Beyond these areas, phage-derived antibodies have proven effective in treating ophthalmic diseases (ranibizumab for neovascular age-related macular degeneration and diabetic retinopathy)^[113]^, infectious diseases (raxibacumab for inhalation anthrax)^[114,115]^, and hereditary angioedema (lanadelumab)^[116]^. Together, these examples highlight the broad therapeutic impact of phage display biologics. Phage display also facilitates target discovery. For instance, Pavoni *et al.* developed cDNA phage libraries from breast cancer lines (MCF-7, MDA-MB-468), identifying eight novel tumor-associated proteins^[117]^. Among these, two showed frequent upregulation in breast cancer, and one, the T7-1 antigen, displayed strong reactivity with patient sera, marking it as a promising therapeutic target^[117]^.

The technology has further transformed nanobody (Nb) development by exploiting its capacity to present highly diverse libraries (> 10^11^ unique clones). This enables the efficient selection of compact, thermostable, single-domain antibodies with high target specificity. A notable example is the isolation of the high-affinity PD-L1-specific Nb20, which penetrates the tumor stroma. When combined with DC/tumor fusion vaccines, Nb20 enhanced T cell infiltration and anti-angiogenic activity, significantly inhibiting the growth of NSCLC, HCC, and TSCC xenografts while extending survival^[118]^. Similarly, researchers have identified 10 anti-B7-H3 nanobodies from dromedary VHH libraries. CAR-T cells engineered with the B12 nanobody exhibited potent efficacy against pancreatic cancer and neuroblastoma models, significantly improving survival outcomes^[119]^. Collectively, these advancements highlight the expanding role of phage display in modern drug development, spanning diverse therapeutic approaches.

### Phage display in drug delivery

Phage display technology serves as a valuable tool for drug delivery, enhancing targeting precision and accelerating therapeutic effects. Through directed evolution, researchers have engineered phage capsids that display bicyclic RGD4C ligands, which specifically bind to αvβ3/αvβ5 integrins overexpressed on tumor cells and angiogenic endothelial cells within the tumor microenvironment^[120,121]^. This selective binding facilitates efficient drug delivery to both malignant cells and their supporting vasculature. Biodistribution studies in tumor-bearing mice and in domestic dogs with spontaneous cancers have confirmed the high specificity of RGD4C-modified phage vectors, which exhibit minimal off-target transgene delivery. Beyond tumor targeting, phage display has also enabled blood-brain barrier (BBB) penetration, as demonstrated by Wu *et al.*, who utilized this technique to identify nanoparticles that target the transferrin receptor (TfR), thereby enabling brain-specific drug delivery^[122]^.

Further progress has been made with Wang *et al.*, who screened an 8-mer landscape phage library and identified a 55-mer peptide specific to MCF-7 breast cancer cells^[123]^. They then fused this peptide to the N-terminus of the phage coat protein pVIII, generating MCF-7-targeted phage fusion proteins that self-assembled with doxorubicin-loaded liposomes. These nanoparticles exhibited enhanced binding to cancer cells and induced more rapid tumor regression compared with non-targeted treatments. Similarly, Xiao *et al.* identified CSP3, a novel peptide that, when conjugated with nanomaterials and chemotherapeutic agents, serves as an effective targeted delivery system for cervical cancer therapy^[124]^. Collectively, these developments underscore the versatility of phage display in improving drug delivery efficiency while reducing systemic toxicity.

## LIMITATIONS AND CHALLENGES OF PHAGE THERAPY

Although phage therapy holds potential against antibiotic-resistant bacteria, its clinical translation faces limitations. The narrow host range of phages restricts their activity to specific bacterial species or strains. This specificity poses a particular challenge in treating polymicrobial infections or infections involving evolving bacterial populations^[125,126]^. Phage cocktails - combinations of multiple phages with complementary host ranges - have been proposed as a solution^[31,127]^. However, their development is hampered by insufficient clinical testing and potential safety concerns, including increased intestinal permeability and endotoxemia observed in murine models^[128]^. Another limitation is the rapid development of bacterial resistance through mechanisms such as surface receptor modification^[129]^ and horizontal gene transfer^[130]^. Reported resistance rates are as high as 80% in intestinal environments, 50% in sepsis models, and 75% in clinical trials^[131]^. Some studies suggest that phage resistance may inadvertently increase bacterial susceptibility to antibiotics (“phage steering”)^[132,133]^. Nevertheless, the ongoing evolutionary arms race between phages and bacteria remains a central challenge for therapeutic applications.

Pharmacological hurdles also limit the effectiveness of phage therapy. Phage stability varies significantly across physiological contexts due to factors such as pH, temperature, and host immune components^[134,135]^. Neutralizing antibodies induced by phage administration represent a major cause of treatment failure, prompting exploration of protective encapsulation or engineered phages as potential solutions. Additionally, phage-mediated bacterial lysis can release endotoxins, potentially triggering severe inflammatory responses or septic shock^[136,137]^. Delivery barriers are particularly pronounced in mucosal environments, where structural components such as mucins and glycoproteins impede phage diffusion and create bacterial refuges^[138]^.

To address these limitations, ongoing research explores strategies such as combining phages with probiotics, antimicrobials, surfactants, or vaccines^[139,140]^.

However, these combination therapies require extensive investigation to optimize efficacy and safety profiles. Equally important is the development of monitoring systems to track the emergence of phage-resistant bacteria and changes in antibiotic susceptibility. Engineered phages designed to minimize endotoxin release or promote bacterial degradation show promise^[136]^, but their clinical translatability remains unverified. Collectively, these challenges highlight the need for further research and the establishment of standardized protocols addressing host specificity, bacterial resistance, pharmacokinetics, and immune responses to ensure both safety and therapeutic efficacy.

## FUTURE PERSPECTIVES ON PHAGE THERAPY

Phage therapy represents a versatile biological platform with substantial potential in antimicrobial and oncological applications. This review summarizes its key characteristics, mechanisms, advantages, and limitations, while highlighting several challenges that must be addressed before widespread clinical adoption.

First, the clinical application of phage therapy faces major pharmacological challenges that demand careful consideration. Although phage properties such as burst size, adsorption kinetics, solubility, life cycle, biofilm penetration, and latency period have been identified as crucial determinants of therapeutic efficacy^[141,142]^, these parameters remain poorly characterized in human clinical settings. Unlike conventional antibiotics, whose pharmacokinetic/pharmacodynamic (PK/PD) relationships are well established, phages exhibit unique PK properties owing to their particulate nature, replication-dependent activity, and morphological diversity, resulting in complex and variable PK profiles. A key distinction lies in their mechanism of action: phage PD effects require targeted delivery to infection sites followed by local amplification, making standard PK/PD assessment in healthy subjects irrelevant. By contrast, antibiotic PK data from uninfected individuals reliably predict PD outcomes. This biological complexity necessitates comprehensive PK/PD characterization to establish optimized dosing regimens that maximize therapeutic efficacy while minimizing resistance.

Second, the development of phage therapy faces significant regulatory challenges due to the absence of specific frameworks governing phage-based products. Current pharmaceutical regulations, designed for standardized drug development, fail to accommodate the personalized nature of phage therapeutics, thereby creating substantial barriers to product identification, manufacturing, and clinical assessment. A robust regulatory system must rest on three pillars: (1) clearly defined safety and quality standards for phage preparations; (2) standardized yet flexible dosing protocols accounting for phage specificity; and (3) explicit liability frameworks for all stakeholders^[143]^. At present, the Drug and Cosmetic Act categorizes phages ambiguously as biological products without distinct classification, lacking both manufacturing quality controls and clinical evaluation criteria. This regulatory vacuum leaves healthcare providers and manufacturers in legal uncertainty, potentially hindering outbreak responses against emerging multidrug-resistant pathogens. Urgent establishment of tailored regulations would provide essential oversight while enabling rapid deployment against public health threats.

Third, the economic evaluation of phage therapy remains underdeveloped despite its theoretical cost advantages over conventional antibiotics. Although phage isolation and development are substantially less expensive than traditional antibiotic production^[144]^, and their self-replicating nature enables potentially curative single-dose regimens^[145]^, these theoretical benefits have not been systematically validated through real-world cost-effectiveness analyses. Furthermore, the absence of standardized regulatory pathways necessitates case-by-case approval, creating time burdens that must be factored into economic assessments. Establishing regionally curated phage banks with pre-characterized isolates could dramatically reduce development timelines and resource requirements. Integrating AI-assisted matching systems with comprehensive phage libraries would further optimize therapeutic phage selection, minimize diagnostic delays and improve clinical outcomes. Collectively, these strategies could transform phage therapy into an economically viable solution for antimicrobial resistance, particularly if implemented at national or regional levels to ensure broad accessibility.

Fourth, the standardization of phage therapeutics remains challenging due to variation among pathogenic strains. At the production level, genetic diversity among target pathogens necessitates customized phage isolation and purification protocols, creating variability in formulation stability and therapeutic potency that complicates clinical trial design and interpretation^[146]^. This reproducibility issue extends to phage susceptibility testing (PST), where the absence of standardized protocols leads to concerning inter-laboratory variability - with discordance rates exceeding acceptable thresholds even within the same facility across different days^[147]^. Addressing these challenges requires integration of bioinformatics and AI to decipher phage-bacteria recognition patterns and interaction dynamics, enabling the development of standardized, modular phage platforms. Equally problematic is the lack of consensus on safety assessment protocols. While some studies merely report the absence of overt toxicity or resistance development^[148]^, comprehensive evaluation requires systematic monitoring of hematological parameters and gastrointestinal microbiome data. As phage therapy transitions toward broader clinical implementation, unified standards for both efficacy and safety testing must be established to ensure therapeutic reliability and enable meaningful cross-study comparisons.

Fifth, the widespread adoption of phage therapy faces significant sociocultural barriers that must be addressed alongside scientific and regulatory challenges. Longstanding reliance on antibiotics, reinforced by their established safety profiles and routine clinical use, fosters patient reluctance despite phage therapy’s potential benefits. Although a comprehensive review of 59 clinical studies has demonstrated the safety and efficacy of phage therapy in treatment-refractory cases over the past two decades, methodological heterogeneity limits confidence in these findings to a low-to-moderate level^[19]^. Moreover, the viral nature of phages carries an inherent stigma, as the term “virus” predominantly evokes disease associations rather than therapeutic potential in public perception. Overcoming these barriers requires multipronged strategies, including targeted public education to clarify mechanisms of action, dissemination of successful treatment outcomes through patient testimonials, and strategic media engagement to reshape societal perceptions. Such initiatives are critical to establishing phage therapy as a clinically and culturally acceptable alternative to conventional antibiotics.

The successful integration of phage therapy into mainstream healthcare will require a multifaceted approach addressing both scientific and implementation challenges. Foundational to this effort is sustained investment in phage research, particularly targeting multidrug-resistant pathogens such as *E. coli*, *K. pneumoniae*, *P. aeruginosa*, and *A. baumannii*, supported by dedicated funding. Parallel development of innovative economic models, such as orphan drug incentives for therapies targeting rare infections like carbapenem-resistant *K. pneumoniae*, will be essential to ensure commercial viability. Effective knowledge translation platforms must also be established to facilitate interdisciplinary collaboration and foster partnerships between academia, clinical practitioners, and industry stakeholders. Above all, the field requires well-designed clinical trials to generate robust evidence regarding therapeutic efficacy, safety, and cost-effectiveness, which are indispensable for overcoming adoption barriers related to unfamiliarity and resistance concerns. By combining scientific innovation, economic strategies, and clinical validation, phage therapy can progress from experimental status to an established component of modern clinical practice.

## CONCLUSION

This review summarizes numerous clinical cases, demonstrating phage therapy’s evolution from a historical curiosity to a clinically feasible strategy against MDR infections and as an oncological tool. Clinically, it has shown efficacy in treating respiratory, trauma-related, bloodstream, urinary tract, and oral infections caused by pathogens such as *P. aeruginosa*, *A. baumannii*, *K. pneumoniae*, and *M. abscessus*, with reported success rates of 50%-70%, particularly in cases where antibiotics fail. Phage therapy’s targeted mechanism preserves commensal microbiota, while self-amplification at infection sites enables low-dose regimens. Furthermore, phage-antibiotic synergy enhances bacterial eradication and mitigates the development of antibiotic resistance. In oncology, phage display enables high-throughput screening of tumor-targeting peptides and antibodies, expediting the development of tumor diagnostics, drug delivery systems, and novel therapeutics.

Despite this progress, significant challenges remain. Major obstacles include narrow host specificity, rapid bacterial resistance, immunogenicity (e.g., neutralizing antibody production), and limited biofilm penetration. Implementation barriers include the lack of standardized PK/PD models, frameworks for personalized therapy, and scalable production methods. Additionally, phage-induced endotoxin release risks inflammatory complications, and public skepticism toward bacteriophage therapeutics persists. To address these issues, standardized protocols for PK/PD assessment, phage susceptibility testing, and safety monitoring (e.g., endotoxin/immunogenicity screening) must be established. Concurrently, artificial intelligence may facilitate host range prediction, resistance forecasting, and phage cocktail optimization. Regulatory standardization is also urgently needed, requiring institutions to implement phage-specific quality control measures, dosage guidelines, and frameworks to support both compassionate use and commercial development. In conclusion, phage therapy represents a transformative and scalable platform for combating antimicrobial resistance and advancing precision oncology. Although challenges related to specificity, stability, and regulation still exist, coordinated efforts across scientific research, clinical practice, and policy can unlock its full potential.

## DECLARATIONS

### Copyright

© The Author(s) 2025.
